# Identification of an Antimicrobial Agent Effective against Methicillin-Resistant *Staphylococcus aureus* Persisters Using a Fluorescence-Based Screening Strategy

**DOI:** 10.1371/journal.pone.0127640

**Published:** 2015-06-03

**Authors:** Wooseong Kim, Annie L. Conery, Rajmohan Rajamuthiah, Beth Burgwyn Fuchs, Frederick M. Ausubel, Eleftherios Mylonakis

**Affiliations:** 1 Division of Infectious Diseases, Rhode Island Hospital, Alpert Medical School of Brown University, Providence, Rhode Island, United States of America; 2 Department of Molecular Biology, Massachusetts General Hospital, Harvard Medical School, Boston, Massachusetts, United States of America; The Scripps Research Institute and Sorrento Therapeutics, Inc., UNITED STATES

## Abstract

Persisters are a subpopulation of normal bacterial cells that show tolerance to conventional antibiotics. Persister cells are responsible for recalcitrant chronic infections and new antibiotics effective against persisters would be a major development in the treatment of these infections. Using the reporter dye SYTOX Green that only stains cells with permeabilized membranes, we developed a fluorescence-based screening assay in a 384-well format for identifying compounds that can kill methicillin-resistant *Staphylococcus aureus* (MRSA) persisters. The assay proved robust and suitable for high throughput screening (Z`-factor: >0.7). In screening a library of hits from a previous screen, which identified compounds that had the ability to block killing of the nematode *Caenorhabditis* by MRSA, we discovered that the low molecular weight compound NH125, a bacterial histidine kinase inhibitor, kills MRSA persisters by causing cell membrane permeabilization, and that 5 μg/mL of the compound can kill all cells to the limit of detection in a 10^8^ CFU/mL culture of MRSA persisters within 3h. Furthermore, NH125 disrupts 50% of established MRSA biofilms at 20 μg/mL and completely eradicates biofilms at 160 μg/mL. Our results suggest that the SYTOX Green screening assay is suitable for large-scale projects to identify small molecules effective against MRSA persisters and should be easily adaptable to a broad range of pathogens that form persisters. Since NH125 has strong bactericidal properties against MRSA persisters and high selectivity to bacteria, we believe NH125 is a good anti-MRSA candidate drug that should be further evaluated.

## Introduction

A significant challenge in the treatment of bacterial infections has been the appearance of antibiotic-resistant strains as a consequence of mutation or the acquisition of antibiotic resistance genes through horizontal gene transfer, as well as the transient reversible selection of antibiotic-tolerant persister cells during antibiotic therapy in individual patients. Most current antibiotics target essential biosynthetic processes such as DNA replication, protein synthesis, or cell wall synthesis that occur during bacterial growth [1,2]. Antibiotic resistance can be caused by enzymes that degrade or modify the antibiotic, efflux pumps that export the antibiotic, or mutations that modify antibiotic targets [1]. A well-known example of antibiotic resistance is methicillin-resistant *Staphylococcus aureus* (MRSA), which was first identified in the 1960s as a hospital-acquired infection [3], but in recent years has been increasingly prevalent in the general population (community-associated MRSA) [4]. *S*. *aureus* causes approximately 10,800 deaths per year in the United States and approximately 50% of these are due to MRSA [5]. Moreover, although vancomycin is currently used to treat MRSA as an antibiotic of last resort, vancomycin-resistant *S*. *aureus* (VRSA) strains have started to emerge, motivating the urgent development of new antibiotics effective against antibiotic-resistant *S*. *aureus* [6].

In contrast to antibiotic-resistant bacteria such as MRSA, antibiotic-tolerant bacteria, known as persisters, are phenotypic variants that exist as a subpopulation of normal cells. Persisters are non-growing dormant bacteria where the targets for most conventional antibiotics are inactive [7,8]. Persisters were first identified by Bigger in 1944 [9], but the molecular mechanisms underlying persister formation are still only partially understood. Recent studies have shown that toxin-antitoxin (TA) modules play an important role in persister formation [10]. Under specific stresses, antitoxins are degraded and the resulting active toxins inhibit cellular processes, which eventually leads to persister formation [8]. Recent studies have shown that persisters are involved in chronic infections and are responsible for the recalcitrance of chronic infections to antibiotic chemotherapy [11,12]. Importantly, persisters are also responsible for the antibiotic tolerance of biofilms [13], surface-associated microbial communities encapsulated by a self-produced extracellular polymeric matrix, that are involved in up to 65% of bacterial infections in developed countries [14].

The bacterial cell envelope consisting of the bacterial membrane and cell wall is a promising target for novel antibiotics that would potentially be effective against both normal and persister cells. The bacterial cell envelope is essential for cell survival and contains about 30% of bacterial proteins, many of which are essential for survival [15–17]. Indeed, many types of antibiotics that target the cell envelope, including proteins, peptides, and small molecules, have been shown to be efficacious against *S*. *aureus* [17–22]. For instance, lysostaphin and endolysins kill *S*. *aureus* by hydrolyzing peptidoglycan, which results in membrane permeabilization [18–20]. Nisin A, daptomycin and telavancin kill *S*. *aureus* by inducing membrane permeabilization and depolarization [17,22]. Although their specific modes of action are different, the common feature of these agents is that each directly or indirectly induces rapid membrane permeabilization, which correlates with bactericidal activity [20,22,23].

We hypothesized that antibiotic agents inducing rapid permeabilization of cell membranes should be effective drugs against MRSA persisters and could be identified using SYTOX Green, a DNA-binding dye that can readily penetrate and stain cells with compromised membranes, but not live cells with intact membranes [24]. Herein, we describe a fluorescence-based screening assay to identify antimicrobial agents that eradicate MRSA persisters by inducing membrane permeabilization. Using this method, we screened a library of antimicrobial compounds previously identified using a high throughput *C*. *elegans*—MRSA assay [25] and identified NH125 (1-Hexadecyl-2-methyl-3-(phenylmethyl)-1*H*-imidazolium iodide) as a hit compound that kills MRSA persisters and eradicates MRSA biofilms. To our knowledge, this is the first high-throughput screening assay for systematically identifying drugs targeting MRSA persisters.

## Materials and Methods

### Bacterial strains, growth conditions, and persister isolation

Community-acquired methicillin-resistance *S*. *aureus* (CA-MRSA) strain MW2 BAA-170 was obtained from ATCC (Manassas, VA, USA). To isolate persister cells, overnight cultures of MW2 grown in tryptic soy broth (TSB) (BD) at 37^°^C were treated with 10X MIC (20 μg/mL) gentamicin for 4 h [26].

### Antimicrobial agents and chemicals

All antibiotics except for NH125 were purchased from Sigma-Aldrich. NH125 was purchased from Tocris Bioscience. 10 mg/mL stock solutions of all antibiotics except for nisin A were made in DMSO or ddH_2_O. As described in a previous study [27], nisin A (hereafter nisin) stock solutions were prepared in 0.02N HCl at a concentration of 250 μg/ml. Lysostaphin-treated samples were supplemented with 0.1% bovine serum albumin (BSA) to prevent nonspecific adherence to plastic surfaces [28].

### Persister membrane permeability assay

Black, clear-bottom 96-well plates (Corning no. 3904) were filled with 50 μL of PBS/well containing the indicated concentration of antibiotics. To isolate MRSA persisters, 25 mL of an *S*. *aureus* MW2 culture was grown to stationary phase and then treated with gentamicin at 20 μg/mL for 4 h, as described above. Then bacteria were washed 3 times with the same volume of phosphate buffered saline (PBS). The washed cells were diluted to OD_600_ = 0.4 (~2 x 10^8^ CFU/mL) with PBS. SYTOX Green (Molecular Probes) was added to 10 mL of the diluted persister suspension to a final concentration of 5 μM and incubated for 30 min at room temperature in the dark. 50 μL of the persister/SYTOX Green mixture was added to each well of 96-well plates containing antibiotics and fluorescence was measured at room temperature for up to 4 h using a spectrophotometer (SpectraMax M3, Molecular Devices) with excitation and emission wavelengths of 485 and 525 nm, respectively. All experiments were conducted in triplicate.

### Time course assay

Persister cells were prepared by treating 25 mL of an *S*. *aureus* MW2 culture in stationary phase with gentamicin at 20 μg/mL for 4 h, as described above. The isolated persisters were washed 3 times with PBS and diluted to OD_600_ 0.4 (~2 x 10^8^ CFU/mL) with the same buffer. 1 mL of the persister suspension containing 10X MICs of indicated antibiotics was added to the wells of a 2 mL deep well assay block (Corning Costar 3960) and incubated at 37^°^C, with shaking at 225 rpm. At specific times, 50 μL samples were removed, serially diluted, and spot-plated on tryptic soy agar (TSA, BD) plates to enumerate the number of persister cells. These experiments were also conducted in triplicate.

### Compound screening

We previously described a high throughput assay for identifying compounds that block the ability of MRSA strain MW2 to kill *C*. *elegans* animals [25]. This assay was used to screen approximately 85,000 compounds obtained from the Institute of Chemistry and Chemical Biology, Harvard Medical School. Among the hits obtained in this screen, which will be described in detail in a separate publication, 101 compounds with antimicrobial properties against MRSA were chosen for a secondary screen to identify compounds that kill persister cells on the basis of their ability to induce permeabilization of MRSA membranes. A black, clear-bottom 384-well plate (Corning no. 3712) was filled with 20 μL of PBS containing 20 μg/mL of each compound from the library. 20 μL of the MRSA persister/SYTOX Green mixture prepared as described above was added into each well of the 384-well plate containing compounds. 0.1% dimethyl sulfoxide (DMSO) or 1.25 μg/mL lysostaphin were included in two columns as negative and positive controls, respectively. Fluorescence was measured as described above. To identify hits, a Z-score was calculated from the fluorescence intensity data; Z-score = (x-μ)/σ where x is the fluorescence intensity of each sample, μ is the mean of the sample population, and σ is the standard deviation of the sample population [29]. Samples with a Z-score>3 were considered hits.

### Z’-factor evaluation for assay quality

The robustness of the screening assay was evaluated based on the Z’-factor [30]. Z’-factor = 1-((3σ_p_+3σ_n_)/|μ_p_-μ_n_|) where σ_p_ and σ_n_ are the standard deviations of the positive and negative controls, respectively and μ_p_ and μ_n_ are the means of the positive and negative controls, respectively [30]. 1>Z’-factor ≥0.5 indicates a robust assay [30]. Fluorescence intensity data from a 384-well plate where half of the wells were filled with 0.1% DMSO (negative control) and the remaining wells contained 1.25 μg/mL lysostaphin or 80 μg/mL nisin (positive controls) were used to calculate the Z’-factor.

### Minimal inhibitory concentration (MIC) assay

The MICs of antibiotics were determined by the standard micro-dilution method recommended by the Clinical and Laboratory Standards Institute [31]. The assay was conducted in triplicate.

### Biofilm persister viability assay

An overnight culture of cells was diluted 1:200 with TSB supplemented with 0.2% glucose and 3% NaCl [32]. A 13 mm diameter Millipore mixed cellulose ester membrane was placed at the bottom of each well of a 12-well plate (Falcon 353043). 1 mL of the diluted culture was added to each well and incubated statically at 37^°^C for 24 h. To remove planktonic cells, the membranes were washed 3-times with PBS and transferred to a new 12-well plate. 1 mL of PBS with 10X MICs of antibiotics was added to each well and the plate was incubated statically at 37^°^C for 24 h. The membranes were washed 3 times with PBS, placed in 1 mL PBS, and sonicated in an ultrasonic bath (Fisher Scientific FS 30) for 10 min. The sonicated samples were serially diluted with PBS in a 96-well plate. The diluted samples were spot-plated on TSA plates and incubated at 37°C overnight. The experiment was conducted in triplicate.

### Biofilm disassembly assay

An overnight culture of *S*. *aureus* MW2 was diluted 1:200 with TSB supplemented with 0.2% glucose and 3% NaCl [32]. 100 μL of the diluted culture was added to each well of a U-bottomed 96-well microtiter plate (Falcon 353077). After 48 h of incubation at 37^°^C, the microtiter plate was washed 3 times with sterile water. 100 μL of PBS including the indicated concentrations of antibiotics were added to each well and incubated at 37^°^C for 24 h. After washing 3 times with water, the biofilm in each well was stained with 0.1% crystal violet (Sigma) for 15 min at room temperature. The plate was washed 3 times with water and then dried. The crystal violet stain was solubilized with 125 μL of 95% ethanol for 15 min. 100 μL of solubilized crystal violet from each sample well was transferred to a new flat-bottomed 96-well microtiter plate and the amount of biofilm was determined by measuring the OD_590 nm_ with a spectrophotometer. The experiment was conducted in triplicate.

## Results

### Isolation of MRSA persisters

**Fig 1:**
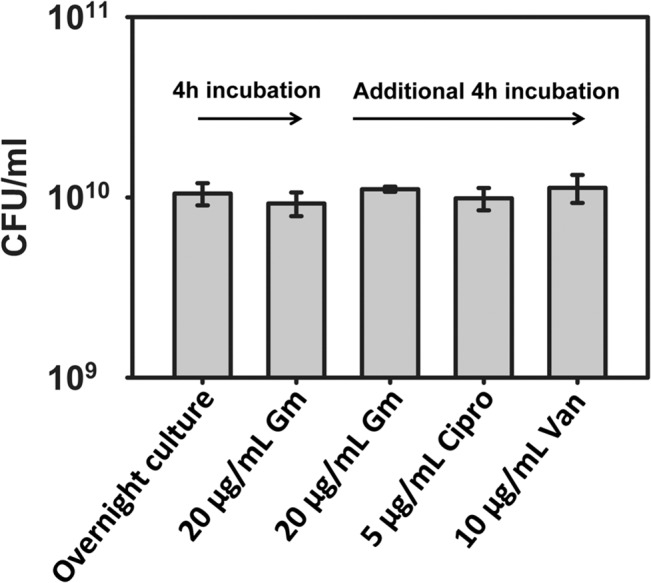
Isolation of MRSA persisters. An MRSA overnight culture was treated with 10X MIC (20 μg/mL) gentamicin for 4 h and the titer of viable cells was determined. After the 4 h treatment with gentamicin, the culture was treated with additional antibiotics at the indicated concentrations (10X MIC) for an additional 4 h, followed by once again determining the titer of viable cells. Results are shown as means ± s.d.; n = 3. Gm: gentamicin, Cipro: ciprofloxacin, Van: vancomycin.

In previous studies, persisters have been isolated from antibiotic-susceptible cell populations by treating stationary phase cultures with a large dose of an antibiotic for 4 h [26,33]. To generate MRSA persisters, *S*. *aureus* MW2 was grown to stationary phase and then treated with 20 μg/mL (10X MIC) gentamicin for 4 h [26]. The concentration of cells in stationary phase was ~10^10^ CFU/mL and, after treating with 20 μg/mL gentamicin for 4 h, the cell viability was not decreased (Fig 1). To determine whether these cells are tolerant to other antibiotics in addition to gentamicin, we treated the gentamicin-tolerant cells with an additional dose of 10X MIC gentamicin, or with ciprofloxacin (DNA synthesis inhibitor) or 10X MIC vancomycin (cell wall synthesis inhibitor) for an additional 4 h. No decrease in viability was observed after treating the gentamicin-tolerant cells with any of these antibiotics (Fig 1). Moreover, these gentamicin-tolerant cells do not show a further decrease in viability after treating with 100X MIC gentamicin, ciprofloxacin or vancomycin for 4 h (S1 Fig). These results indicated that essentially all of the *S*. *aureus* MW2 cells in a stationary phase culture are in a persistent state, which is consistent with previous studies showing that stationary-phase *S*. *aureus* cells are persisters [26,33,34].

### SYTOX Green assay to identify compounds that kill persister cells

Several potent antibiotics such as lysostaphin, nisin, and HT61 confer antimicrobial activity by damaging the cell wall or membrane, which both directly and indirectly causes membrane permeabilization [20,22,35]. Because both actively dividing cells and persisters depend on an intact cell envelope for viability, as noted above, we reasoned that antimicrobial compounds that cause membrane permeabilization would be good candidates for potential drugs effective against MRSA persisters. In order to identify such compounds, we developed an assay using the reporter dye SYTOX Green that is only taken up by cells with permeabilized membranes and shows >500-fold signal enhancement upon binding to DNA [24].

**Fig 2:**
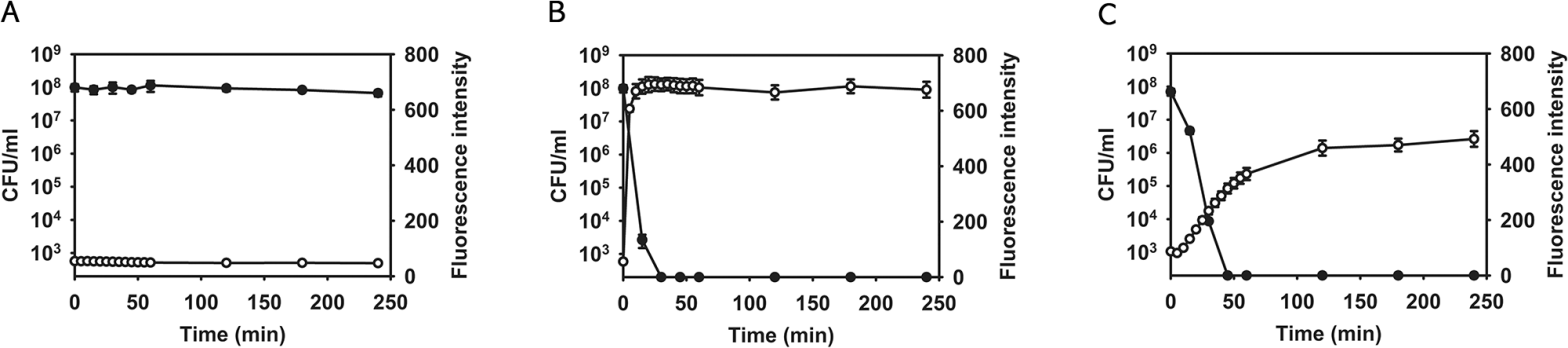
Lysostaphin and nisin kill MRSA persisters by inducing membrane permeabilization. MRSA persisters were treated with 0.1% DMSO (A), 10X MIC lysostaphin (B), or 10X MIC nisin (C). Membrane permeabilization (open circles) was measured spectrophotometrically by monitoring the uptake of SYTOX Green (excitation wavelength of 485 nm and an emission wavelength of 525 nm). Colony forming unit counts of persisters (solid circles) were measured by serial dilution and plating on TSA plates. The data points on the x-axis are below the level of detection (2x10^2^ CFU/mL). Results are shown as means ± s.d.; n = 3.

As a proof-of-concept, we measured membrane permeability and viability of MRSA persister cells after treatment with lysostaphin or nisin. As expected, both lysostaphin and nisin did not only induce membrane permeabilization, but also killed persisters, as measured by CFU counts, at a rate that was directly proportional to their concentration (Fig 2 and S2 Fig). In contrast, traditional antibiotics such as gentamicin, vancomycin, and ciprofloxacin at 10X MIC did not cause membrane permeabilization or cell death (S3 Fig). In addition, we found a strong correlation between membrane permeability as measured by the SYTOX Green assay and viability (Fig 2 and S2 Fig). A correlation between membrane permeability and cell death was also observed with an exponential-phase *S*. *aureus* culture treated with lysostaphin [20] or nisin [36], suggesting that their bactericidal activities correlate with membrane permeabilization and are the same in both growing and persister cells. These results support the hypothesis that antimicrobial agents that kill MRSA persisters by inducing membrane permeabilization can be identified using SYTOX Green.

### High-throughput MRSA persister cell screen

**Fig 3:**
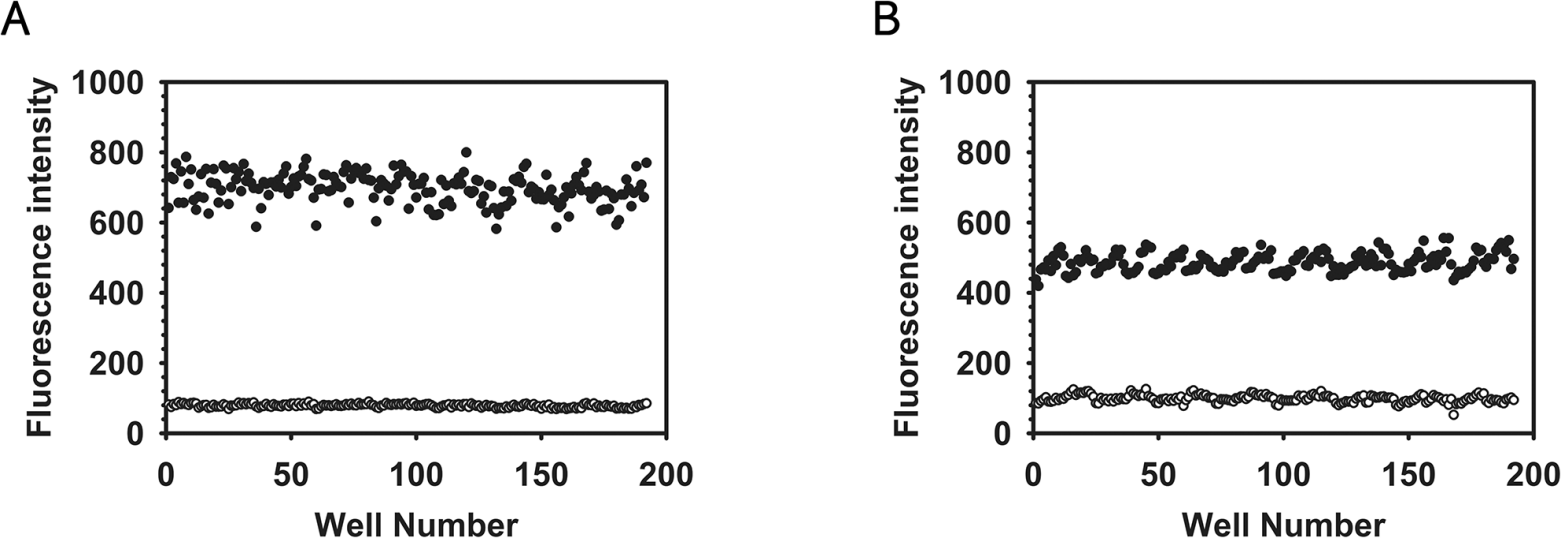
Validation of SYTOX Green assay robustness. To test the robustness of the SYTOX Green assay, the Z’-factor was calculated from fluorescence intensity data from a 384-well plate where half of the wells contained 0.1% DMSO (negative control, open circles) and the remaining wells contained 10X MIC lysostaphin (A) or 10X MIC nisin (B) (positive controls, solid circles). Fluorescence was measured with an excitation wavelength of 485 nm and an emission wavelength of 525 nm after incubation in the dark for 1 h. The Z’-factors for each assay were 0.767 (A) and 0.712 (B).

The assay for identifying anti-persister drugs using SYTOX Green was adapted for HTS in 384-well microtiter plate format. The volume for the assay was adjusted to 40 μL per well, and fluorescence intensity was measured 1 h after compounds were added, based on the expectation that effective compounds would induce rapid membrane permeabilization (Fig 2B and 2C). To evaluate the robustness and reproducibility of the assay, we determined the Z’-factor, a statistical parameter of assay quality as noted in the Materials and Methods [30]. The Z’-factor was calculated from fluorescence intensity data from a 384-well plate in which half of the wells were filled with 0.1% DMSO (negative control) and the remaining wells contained 10X MIC of lysostaphin or 10X MIC of nisin (positive controls). The Z’-factors calculated using lysostaphin and nisin were 0.767 and 0.712, respectively (Fig 3), which indicated that this assay is robust and suitable for large-scale, high throughput screening.

### Pilot screen and identification of a compound that kills MRSA persisters

We previously screened 85,000 compounds obtained from the Institute of Chemistry and Cell Biology (ICCB), Harvard Medical School, using a *C*. *elegans-*MRSA assay [25] (manuscript in preparation) in order to identify anti-MRSA agents that are able to prolong the lifespan of *C*. *elegans* infected with MRSA. One of the advantages of this *C*. *elegans*-based screening strategy is the ability to simultaneously assess toxicity and efficacy [25,37–40]. 101 anti-MRSA agents that prolonged the life of MRSA-infected nematodes, identified using the *C*. *elegans-*MRSA assay, were screened at 10 μg/mL utilizing the SYTOX Green permeability assay. To identify hits, Z-scores were calculated from the fluorescence intensity data.

**Fig 4:**
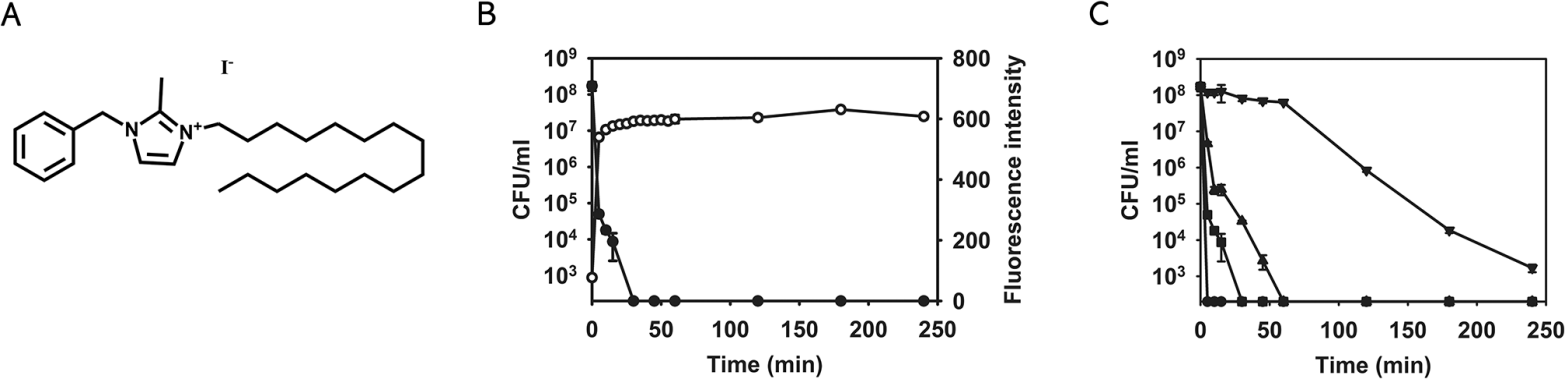
NH125 kills MRSA persisters by inducing membrane permeabilization. (A) The chemical structure of NH125. (B) MRSA persisters were treated with 10 μg/mL NH125. Membrane permeabilization (open circles) was measured spectrophotometrically by monitoring the uptake of SYTOX Green (excitation wavelength of 485 nm and an emission wavelength of 525 nm). Colony forming unit counts of persisters (solid circles) was measured by serial dilution and plating on TSA plates. (C) MRSA persisters were treated with several concentrations of NH125: 10X MIC (20 μg/mL, circles), 5X MIC (10 μg/mL, squares), 2.5X MIC (5 μg/mL, triangles), and 1X MIC (2 μg/mL, inverted triangles). Colony forming unit counts of persisters was measured by serial dilution and plating on TSA plates. The data points on the x-axis are below the level of detection (2x10^2^ CFU/mL). Results are shown as means ± s.d.; n = 3.

Among the 101 compounds, NH125 (1-Hexadecyl-2-methyl-3-(phenylmethyl)-1*H*-imidazolium iodide) was identified as a hit with a Z-score of 10.61 (Fig 4A). NH125 is known to be an antibiotic and has been shown to inhibit bacterial histidine kinases and eukaryotic elongation factor 2 kinase [41–43]. Consistent with previous work [41,42], the MIC of NH125 against *S*. *aureus* MW2 is ~2 μg/mL (Table 1). As shown in Fig 4B, 10 μg/mL NH125 not only induced rapid membrane permeabilization, but also resulted in a dramatic decrease in the viability of MRSA persisters. To confirm the correlation between membrane permeabilization and viability, we tested 5 sample compounds also identified in the *C*. *elegans*—MRSA screen that did not have activity in the SYTOX Green assay and found that they did not decrease CFU counts (data not shown). Next, we assessed the killing efficiency of NH125 on MRSA persisters at various NH125 concentrations. 2.5X MIC (5 μg/mL) NH125 was sufficient to completely eradicate MRSA persisters within 1 h, and 1X MIC (2 μg/mL) killed 99.999% of MRSA persisters within 4 h (Fig 4C).

**Table 1:** Minimal inhibitory concentration (MIC) against *S*. *aureus* MW2.

Compound	MIC (μg/mL)
Gentamicin	2
Vancomycin	1
Ciprofloxacin	0.5
Lysostaphin	0.125
Nisin	8
NH125	2

One of the advantages of the SYTOX Green screening method is that positive hits that kill persister cells by permeabilizing the membrane should also be able to kill non-persister cells. Like lysostaphin [20] and nisin [22], the correlation between viability and membrane permeability was also observed in growing MRSA treated with 10 μg/ml NH125 (S4 Fig).

### NH125 kills and disrupts MRSA biofilms

**Fig 5:**
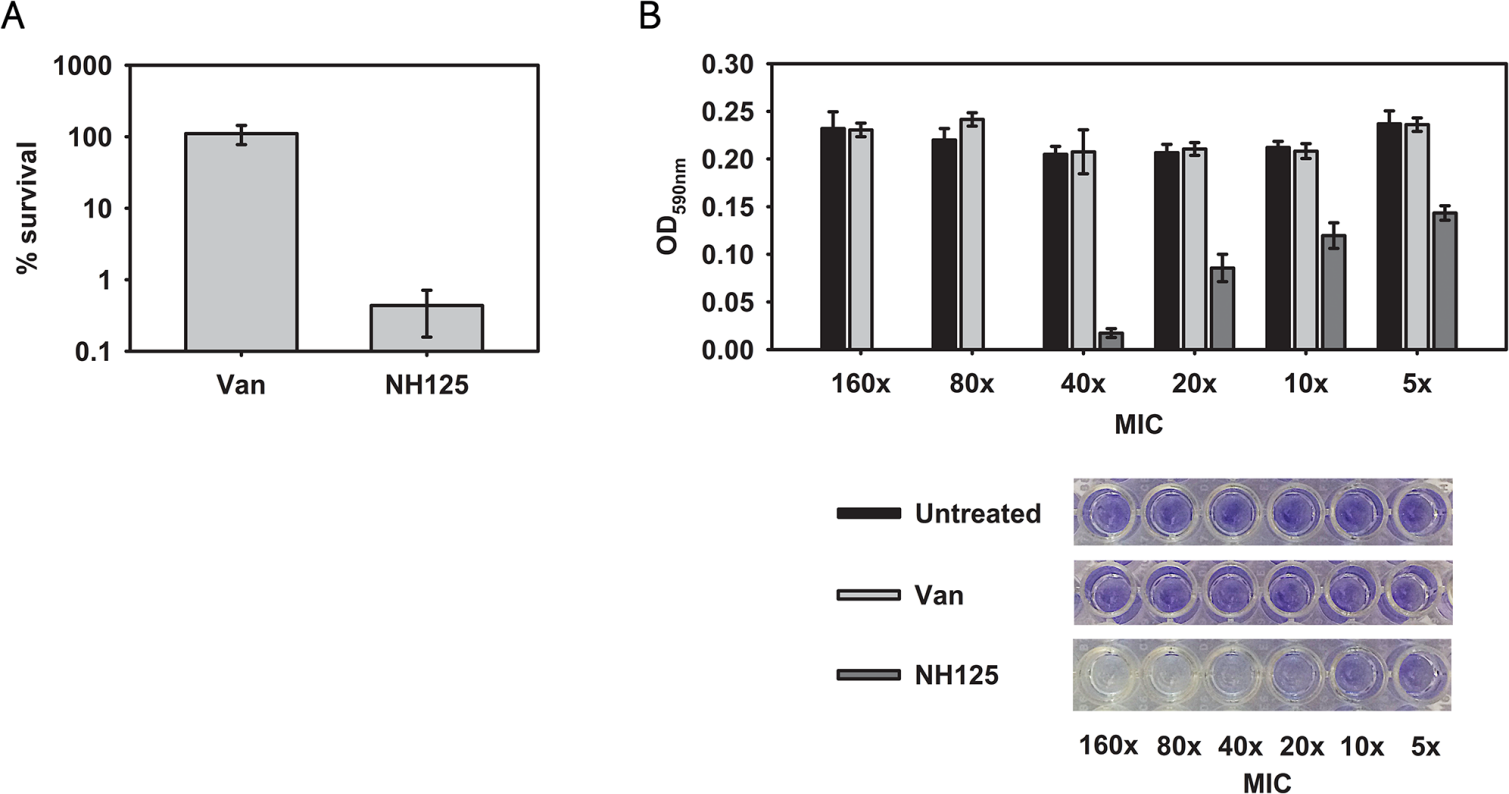
NH125 eradicates MRSA biofilms. (A) MRSA biofilms formed on 13 mm cellulose ester membranes for 24 hours were treated with 10X MIC of vancomycin (Van) or NH125 for 24 h. Survival was measured by comparing the number of viable cells in biofilms between non-treated and treated samples. (B) MRSA biofilms grown in a 96-well microtiter plate for 48 h were treated with the indicated concentration of vancomycin or NH125 for 24 h. The remaining biofilms were stained with 0.1% crystal violet dissolved with 95% ethanol and OD_590 nm_ was measured. Results are shown as means ± s.d.; n = 3.

Since persisters in biofilms are known to be responsible for antibiotic tolerance of biofilms [44,45], we reasoned that NH125 would be effective in eradicating biofilms. First, we assessed the ability of NH125 to kill MRSA cells in biofilms. For these experiments, MRSA biofilms statically cultured on 13 mm mixed cellulose membranes for 24 h at 37^°^C were treated with vancomycin or NH125 at 10X MIC for 24 h. Individual cells were freed from the biofilm matrix by sonication and cell viability was measured with CFU counts. As with planktonic persisters, vancomycin was unable to kill MRSA cells in biofilms (Fig 5A). In contrast, NH125 killed over 99% of MRSA biofilm cells at 20 μg/mL (Fig 5A).

In addition to killing cells within biofilms, we assessed the ability of NH125 to disassemble biofilm biomass. MRSA biofilms formed in a 96-well microtiter plate for 48 h at 37^°^C were treated with various concentrations of vancomycin or NH125 (5X MIC to 160X MIC) for 24 h. The entire biofilm biomass was quantified using crystal violet, a cationic dye that stains all components of biofilms including cells and EPS [46,47]. Up to 160X MIC of vancomycin was unable to disrupt MRSA biofilms (Fig 5B). However, 10X MIC of NH125 removed 50% of biofilm biomass, and 80X MIC of NH125 completely disassembled the biofilm (Fig 5B). These results indicate that NH125 is able to effectively penetrate the EPS matrix, a protective “shield” around biofilms, and kill persister cells as well as disrupting established biofilms.

## Discussion

*S*. *aureus* is one of the most dangerous Gram-positive pathogens in the context of human health. Up to 30% of individuals are carriers of *S*. *aureus*, which can cause a range of infectious diseases from acute to chronic infections in both healthy individuals and immunocompromised patients such as those with cancer and AIDS [4,48–54]. MRSA exhibiting resistance to commonly prescribed beta-lactam antibiotics is increasingly prevalent in hospitals as well as the community at large and has become an important public health problem [55]. Since MRSA can form persisters and is associated with chronic infection [56], development of drugs against MRSA persisters would be a significant advance in the treatment of MRSA infections.

To date, a variety of strategies have been used to kill persisters formed by *S*. *aureus* and MRSA. The first strategy was to facilitate the uptake of aminoglycosides into MRSA persisters. Although most biosynthetic processes that are targets for antibiotics are minimized in persisters, proteins are synthesized at a low level, and therefore, aminoglycosides can be effective against persisters [26,57]. However, the uptake of aminoglycosides is minimal in persisters due to the inactive state of transport mechanisms [26,57,58]. Allison *et al*. reported that metabolites such as glucose, mannitol, or fructose can make *S*. *aureus* persisters aminoglycoside-susceptible by increasing the proton motive force of persister cell membranes [26]. Schmidt *et al*. engineered tobramycin by attachment of 12 amino acids, which promotes the uptake of tobramycin and subsequent killing of *S*. *aureus* persisters [57]. A second strategy was to induce protein degradation in persister cells by activating a protease. Conlon *et al*. identified ADEP4 that kills MRSA persisters by activating the ClpP protease, which subsequently leads to non-specific degradation of over 400 proteins including several that are essential for bacterial survival [34]. The third strategy was to directly attack structural components of the cell envelope such as the membrane or cell wall. As reviewed in Hurdle *et al*., many membrane active agents such as HT61 are known to kill *S*. *aureus* persisters [17]. In addition, Gutiérrez *et al*. reported that the phage endolysin LysH5 is able to eradicate *S*. *aureus* persisters by hydrolyzing peptidoglycan [59]. However, a screening method for systematically identifying drugs targeting MRSA persisters has not previously been developed.

Based on the fact that cell envelope-targeting agents can directly and indirectly cause membrane permeabilization, which is correlated with bactericidal activity [20,22,23], we devised a HTS using SYTOX Green to discover new antimicrobial agents that are effective against MRSA persisters. Statistical evaluation of assay robustness showed that the assay is suitable for a large-scale screen. The SYTOX Green assay should also be broadly applicable for screens for drugs effective against persisters of other multidrug-resistant pathogens since SYTOX Green shows selective permeability in both Gram-positive and Gram-negative bacteria [24]. For example, we found a correlation between membrane permeability and viability of *E*. *coli* persisters after treatment with polymyxin B [60], a membrane active antibiotic effective against Gram-negative bacteria (S6B Fig). By conducting a pilot screen with 101 anti-MRSA agents from a previously conducted *C*. *elegans*-MRSA screen for compounds that block the ability of MRSA to kill the nematodes, we identified NH125, which has low toxicity but strong antimicrobial property against MRSA persisters.

In our “proof of principle” screen of candidate antibiotics, we identified NH125 as a compound that is able to kill MRSA persisters. NH125 is already known to have antimicrobial properties with antibiotic activity (MIC 0.39–3.12 μg/mL) against drug-resistant Gram-positive bacteria, such as oxacillin-resistant *S*. *aureus*, penicillin G-resistant *Streptococcus pneumoniae*, and vancomycin-resistant *Enterococcus faecalis* [41]. A recent study revealed that NH125 inhibits histidine kinases of bacterial two component signal transduction systems (TCS) by inducing non-specific aggregation of the histidine kinases [61]. NH125 has also been investigated as a potential antineoplastic drug with activity against eukaryotic elongation factor 2 kinase (eEF2K), which was shown to be due to nonspecific colloidal aggregation [62]. Based on the known kinase aggregation activity of NH125 [61,62], a possible mechanism by which NH125 kills persister cells may be by binding and aggregating kinases on the MRSA cell membrane, causing structural changes that result in membrane permeabilization and death.

Although many membrane-damaging agents have excellent anti-MRSA properties, including a low MIC, rapid killing rate, and low probability for developing resistance, they have a tendency to cause toxicity in mammals [63,64]. However, our starting library of 101 anti-MRSA agents identified using the *C*. *elegans*-MRSA HTS was expected to be enriched for nontoxic compounds, since the endpoint of the assay is enhanced survival of the nematodes in the presence of MRSA. In fact, the invertebrate nematode *C*. *elegans* has been used as a model for assessing toxicity of many chemicals, including heavy metals, environmental pollutants, organic solvents, and neurotoxins [65–67]. Moreover, many studies have shown a strong correlation in toxicity between *C*. *elegans* and rodents [68,69]. In the *C*. *elegans*-MRSA HTS, 7.5 μg/mL NH125 kills MRSA but shows no toxicity to *C*. *elegans* (S7 Fig). Consistent with this result, up to 30 μM (15.8 μg/mL) NH125 has been reported to be nontoxic to sea urchin eggs [70]. Because of low toxicity, the kinase inhibitory activity of NH125 has been intensively studied in human cells and mice [43,71–74]. Considering 2 μg/mL of NH125 (equivalent to 1X MIC) kills 99.999% of MRSA persister cells within 4 h, we believe NH125 is a good candidate drug that warrants further studies as a therapeutic against MRSA persisters.

In summary, we have devised a fluorescence-based HTS for identifying drugs that eradicate MRSA. Using this screening method, we identified NH125, a compound that effectively kills MRSA persisters by inducing rapid membrane permeabilization but has selectivity to bacteria. Furthermore, NH125 kills MRSA persisters in biofilms and eradicates established MRSA biofilms. The screening method we developed can be used as a large-scale screening platform for antibiotic drug discovery against persisters of a broad range of pathogens. NH125, and/or derivatives of this molecule, warrant further evaluation as antibiotics for treatment of persistent or chronic infections.

## Supporting Information

S1 Fig Isolated MRSA persisters are tolerant to 100X MIC conventional antibiotics. A MRSA overnight culture was treated with 10X MIC (20 μg/mL) gentamicin for 4 h and the titer of viable cells was determined. After the 4 h treatment with gentamicin, the culture was treated with additional antibiotics at the indicated concentrations (100X MIC) for an additional 4 h, followed by once again determining the titer of viable cells. Results are shown as means ± s.d.; n = 3. Gm: gentamicin, Cipro: ciprofloxacin, Van: vancomycin.
(TIFF)

S2 Fig Lysostaphin and nisin kill MRSA persisters by inducing membrane permeabilization in a dose-dependent manner. MRSA persisters were treated with 5X MIC lysostaphin (A), 2.5X MIC lysostaphin (B),1X MIC lysostaphin (C), 5X MIC nisin (D), 2.5X MIC nisin (E), or 1X MIC nisin (F). Membrane permeabilization (open circles) was measured spectrophotometrically by monitoring the uptake of SYTOX Green (excitation wavelength of 485 nm and an emission wavelength of 525 nm). Colony forming unit counts of persisters (solid circles) were measured by serial dilution and plating on TSA plates. The data points on the x-axis are below the level of detection (2x10^2^ CFU/mL). Results are shown as means ± s.d.; n = 3.
(TIFF)

S3 Fig Conventional antibiotics do not kill MRSA persisters or induce membrane permeabilization. MRSA persisters were treated with 10X MIC (20 μg/mL) gentamicin, 10X MIC (10 μg/mL) vancomycin (B), or 10X MIC (5 μg/mL) ciprofloxacin (C). Membrane permeabilization (open circles) was measured spectrophotometrically by monitoring the uptake of SYTOX Green (excitation wavelength of 485 nm and an emission wavelength of 525 nm). Colony forming unit counts of persisters (solid circles) was measured by serial dilution and plating TSA plates. Results are shown as means ± s.d.; n = 3.
(TIFF)

S4 Fig NH125 kills growing MRSA by inducing membrane permeabilization. Growing MRSA was treated with 10 μg/ml NH125. Membrane permeabilization (open circles) was measured spectrophotometrically by monitoring the uptake of SYTOX Green (excitation wavelength of 485 nm and an emission wavelength of 525 nm). Colony forming unit counts of persisters (solid circles) were measured by serial dilution and plating on TSA plates. The data points on the x-axis are below the level of detection (2x10^2^ CFU/mL). Results are shown as means ± s.d.; n = 3.
(TIFF)

S5 Fig Increase of ciprofloxacin concentration up to 1000X MIC does not affect the viability of *E*. *coli* persisters. An *E*. *coli* MG1655 overnight culture was treated with 10X MIC (0.3 μg/mL), 100X MIC (3 μg/ml), or 1000X MIC (30 μg/ml) ciprofloxacin for 4 h and the titer of viable cells was determined. Results are shown as means ± s.d.; n = 3. Gm: gentamicin, Cipro: ciprofloxacin, Van: vancomycin.
(TIFF)

S6 Fig Polymyxin B kills *E*. *coli* persisters by inducing membrane permeabilization. *E*. *coli* MG1655 persisters were treated with 0.1% DMSO (A), 10X MIC (20 μg/ml) polymyxin B (B), 10X MIC (0.3 μg/mL) ciprofloxacin (C), 10X MIC (160 μg/mL) ampicillin (D), or 10X MIC (40 μg/mL) gentamicin (E). Membrane permeabilization (open circles) was measured spectrophotometrically by monitoring the uptake of SYTOX Green (excitation wavelength of 485 nm and an emission wavelength of 525 nm). Colony forming unit counts of persisters (solid circles) were measured by serial dilution and plating on TSA plates. The data points on the x-axis are below the level of detection (2x10^2^ CFU/mL). Results are shown as means ± s.d.; n = 3.
(TIFF)

S7 Fig NH125 has antimicrobial activity against MRSA but no toxicity to *C*. *elegans*. 15 adult worms were transferred in 384-well plates. Each well contained 70 μL media including 70% M9 buffer, 19% sheath solution (Union Biometrica Part no. 300-5101-000), 10% TSB, and 1% DMSO. The bacterial concentration in each well was adjusted to OD_600_ 0.04, and the final concentration of drugs was 7.5 μg/mL. After incubation in a humidified chamber at 25^°^C for 5 days, the worms were washed 8-times with M9 buffer and stained with 0.7 μM SYTOX Orange for staining dead worms. The plates were imaged using an Image Xpress Micro automated microscope (Molecular Devices), capturing both transmitted light and TRITC (535 nm excitation, 610 nm emission) fluorescent images with a 2X objective.
(TIFF)
