# Supplementary figures and images for: Identification of an Antimicrobial Agent Effective against Methicillin-Resistant Staphylococcus aureus Persisters Using a Fluorescence-Based Screening Strategy
Source: PLoS One. 2015 Jun 3;10(6):e0127640. doi: 10.1371/journal.pone.0127640 (PMC4454602; doi:10.1371/journal.pone.0127640)

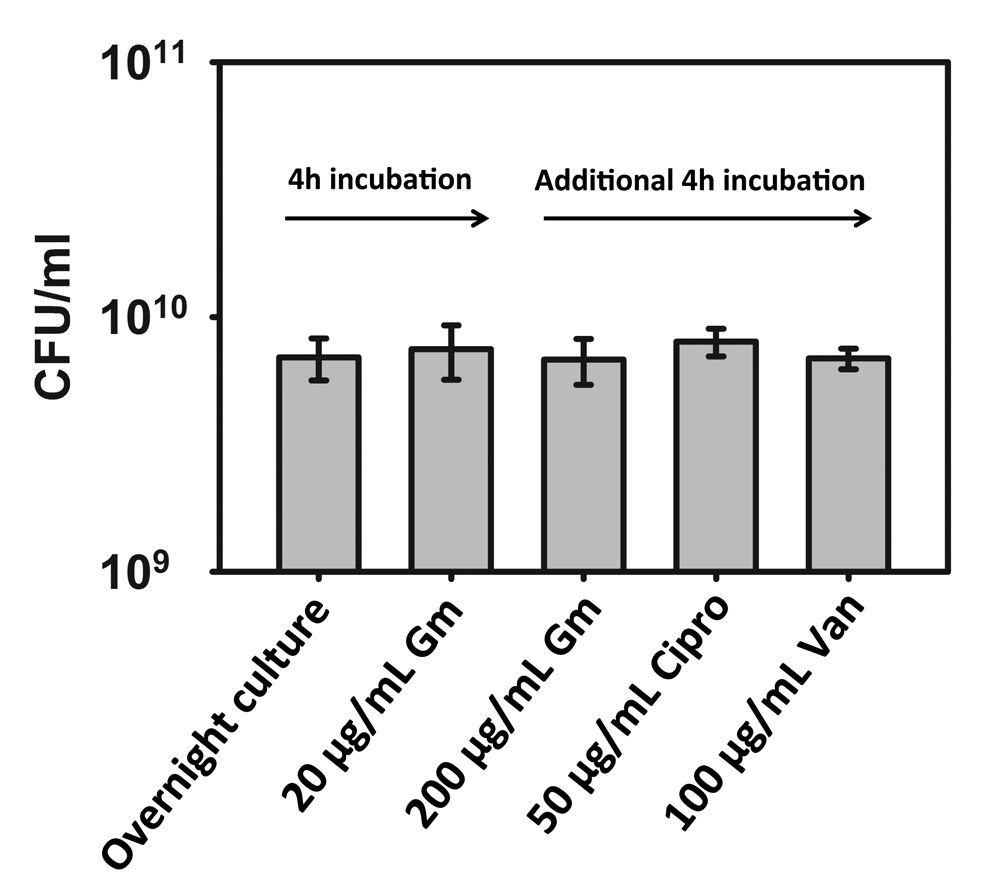

Supplement: S1 Fig — A MRSA overnight culture was treated with 10X MIC (20 μg/mL) gentamicin for 4 h and the titer of viable cells was determined. After the 4 h treatment with gentamicin, the culture was treated with additional antibiotics at the indicated concentrations (100X MIC) for an additional 4 h, followed by once again determining the titer of viable cells. Results are shown as means ± s.d.; n = 3. Gm: gentamicin, Cipro: ciprofloxacin, Van: vancomycin. (TIFF) [file pone.0127640.s001.tiff]

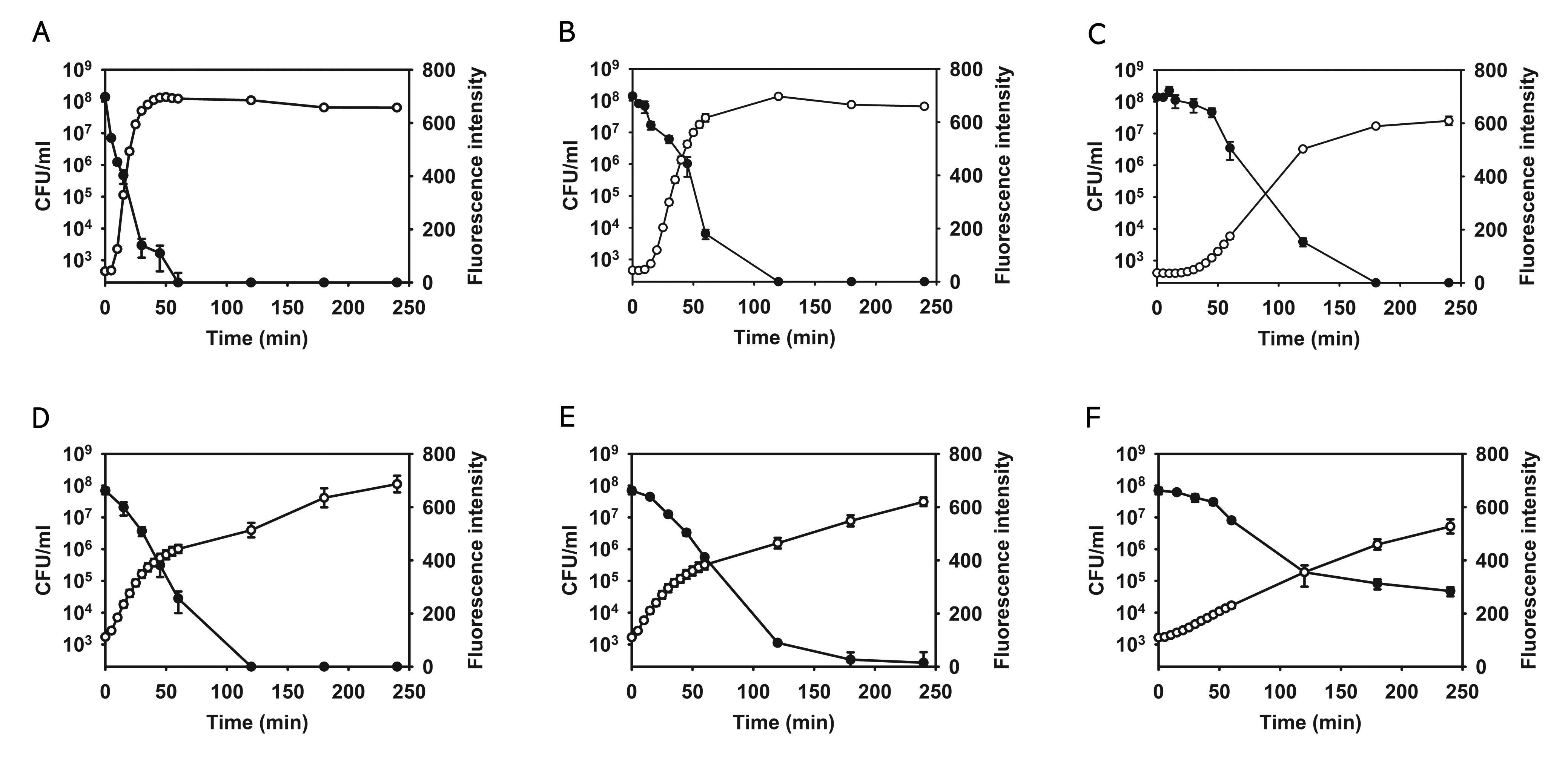

Supplement: S2 Fig — MRSA persisters were treated with 5X MIC lysostaphin (A), 2.5X MIC lysostaphin (B),1X MIC lysostaphin (C), 5X MIC nisin (D), 2.5X MIC nisin (E), or 1X MIC nisin (F). Membrane permeabilization (open circles) was measured spectrophotometrically by monitoring the uptake of SYTOX Green (excitation wavelength of 485 nm and an emission wavelength of 525 nm). Colony forming unit counts of persisters (solid circles) were measured by serial dilution and plating on TSA plates. The data points on the x-axis are below the level of detection (2x10^2^ CFU/mL). Results are shown as means ± s.d.; n = 3. (TIFF) [file pone.0127640.s002.tiff]

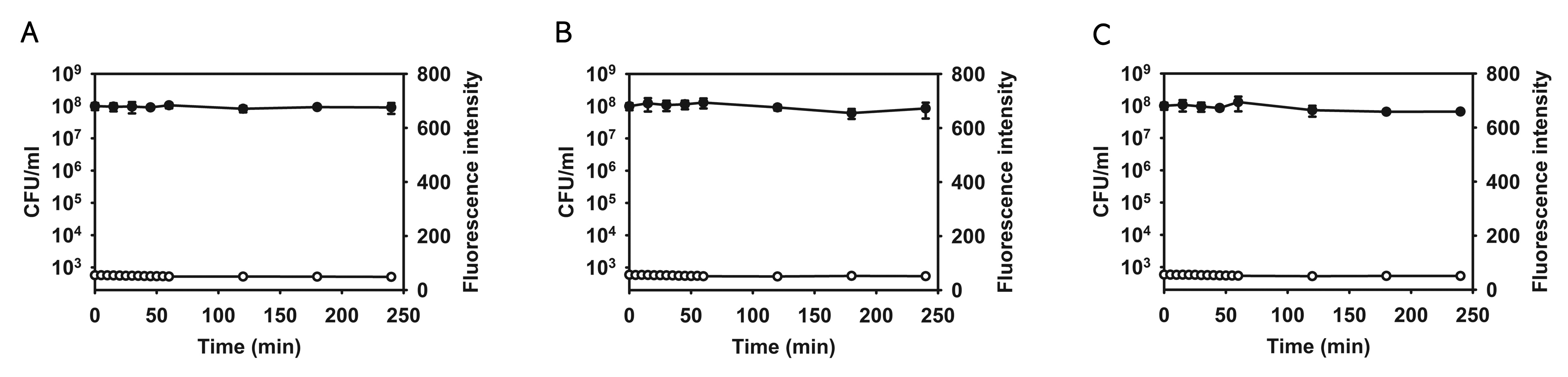

Supplement: S3 Fig — MRSA persisters were treated with 10X MIC (20 μg/mL) gentamicin, 10X MIC (10 μg/mL) vancomycin (B), or 10X MIC (5 μg/mL) ciprofloxacin (C). Membrane permeabilization (open circles) was measured spectrophotometrically by monitoring the uptake of SYTOX Green (excitation wavelength of 485 nm and an emission wavelength of 525 nm). Colony forming unit counts of persisters (solid circles) was measured by serial dilution and plating TSA plates. Results are shown as means ± s.d.; n = 3. (TIFF) [file pone.0127640.s003.tiff]

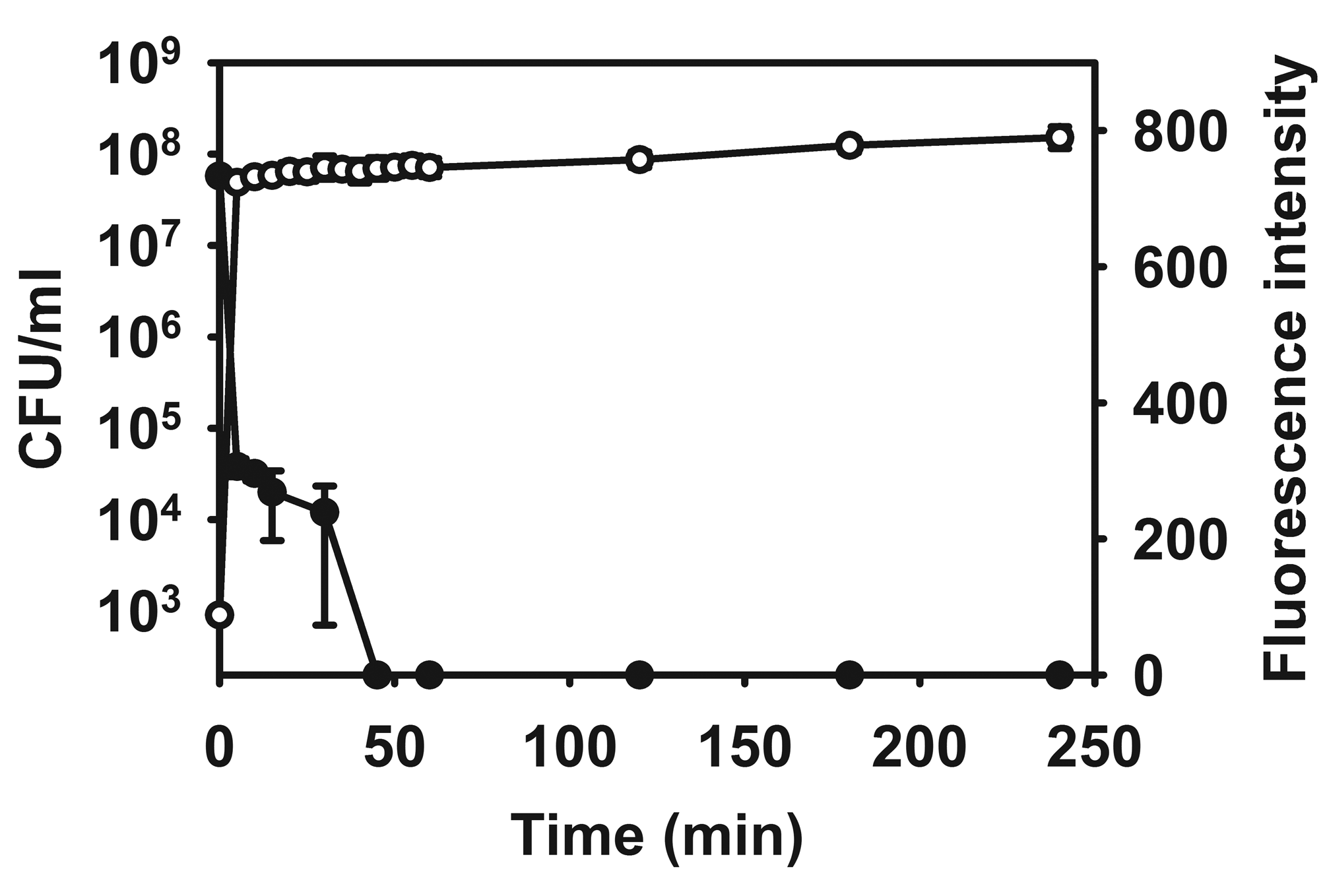

Supplement: S4 Fig — Growing MRSA was treated with 10 μg/ml NH125. Membrane permeabilization (open circles) was measured spectrophotometrically by monitoring the uptake of SYTOX Green (excitation wavelength of 485 nm and an emission wavelength of 525 nm). Colony forming unit counts of persisters (solid circles) were measured by serial dilution and plating on TSA plates. The data points on the x-axis are below the level of detection (2x10^2^ CFU/mL). Results are shown as means ± s.d.; n = 3. (TIFF) [file pone.0127640.s004.tiff]

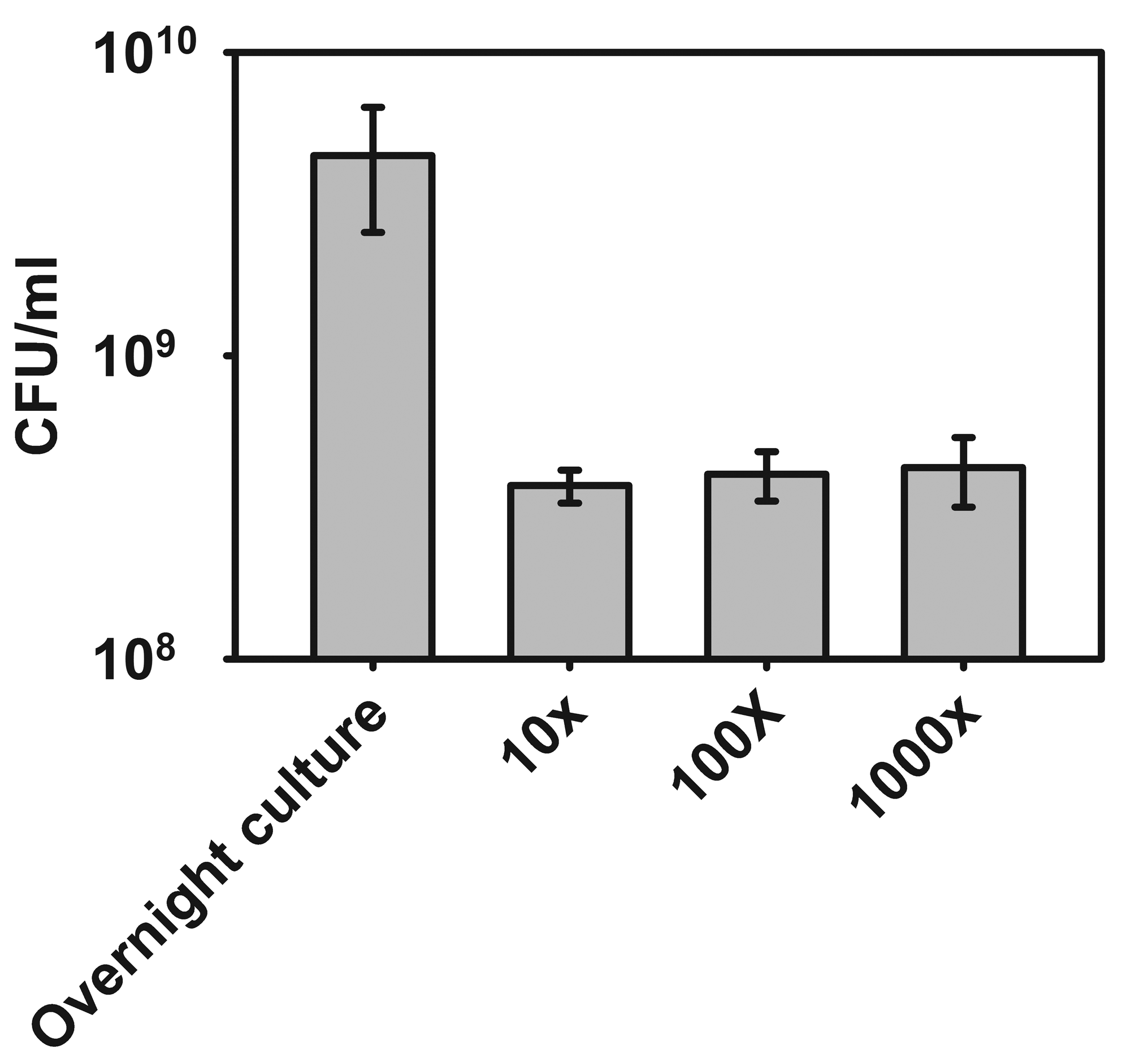

Supplement: S5 Fig — An E. coli MG1655 overnight culture was treated with 10X MIC (0.3 μg/mL), 100X MIC (3 μg/ml), or 1000X MIC (30 μg/ml) ciprofloxacin for 4 h and the titer of viable cells was determined. Results are shown as means ± s.d.; n = 3. Gm: gentamicin, Cipro: ciprofloxacin, Van: vancomycin. (TIFF) [file pone.0127640.s005.tiff]

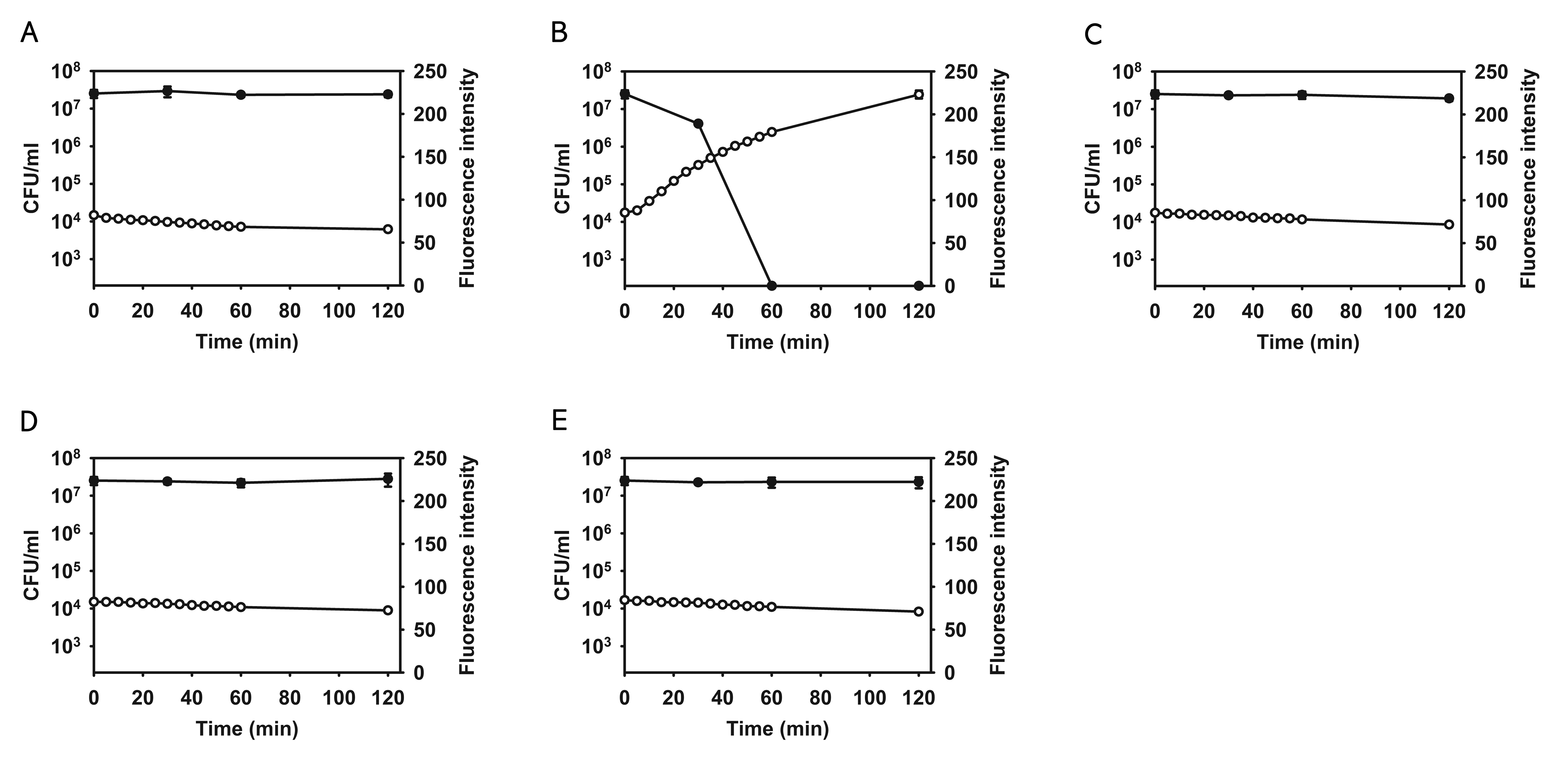

Supplement: S6 Fig — E. coli MG1655 persisters were treated with 0.1% DMSO (A), 10X MIC (20 μg/ml) polymyxin B (B), 10X MIC (0.3 μg/mL) ciprofloxacin (C), 10X MIC (160 μg/mL) ampicillin (D), or 10X MIC (40 μg/mL) gentamicin (E). Membrane permeabilization (open circles) was measured spectrophotometrically by monitoring the uptake of SYTOX Green (excitation wavelength of 485 nm and an emission wavelength of 525 nm). Colony forming unit counts of persisters (solid circles) were measured by serial dilution and plating on TSA plates. The data points on the x-axis are below the level of detection (2x10^2^ CFU/mL). Results are shown as means ± s.d.; n = 3. (TIFF) [file pone.0127640.s006.tiff]

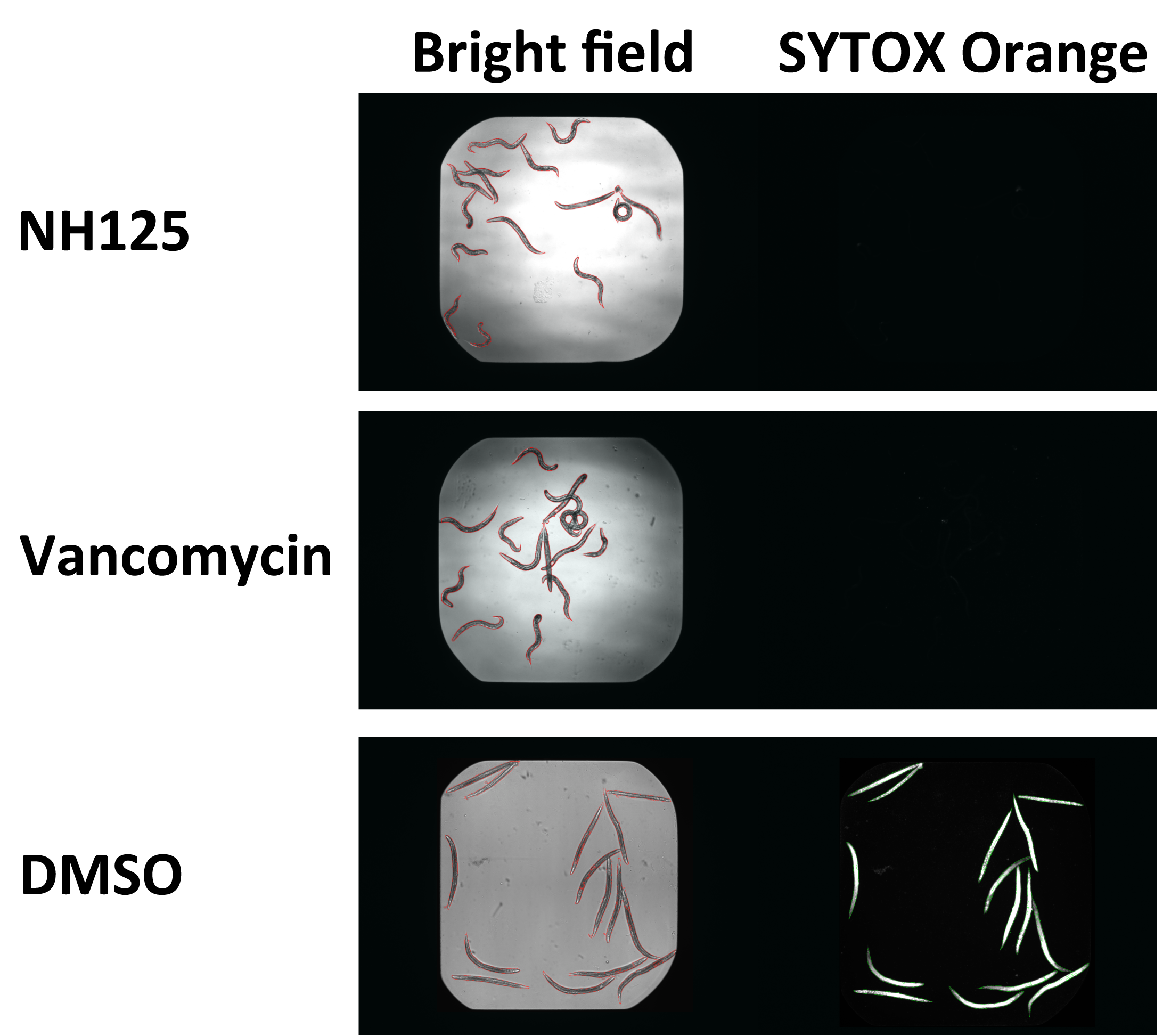

Supplement: S7 Fig — 15 adult worms were transferred in 384-well plates. Each well contained 70 μL media including 70% M9 buffer, 19% sheath solution (Union Biometrica Part no. 300-5101-000), 10% TSB, and 1% DMSO. The bacterial concentration in each well was adjusted to OD600 0.04, and the final concentration of drugs was 7.5 μg/mL. After incubation in a humidified chamber at 25°C for 5 days, the worms were washed 8-times with M9 buffer and stained with 0.7 μM SYTOX Orange for staining dead worms. The plates were imaged using an Image Xpress Micro automated microscope (Molecular Devices), capturing both transmitted light and TRITC (535 nm excitation, 610 nm emission) fluorescent images with a 2X objective. (TIFF) [file pone.0127640.s007.tiff]
